# Fit factor of masks used by Physicians in Clinical Settings

**DOI:** 10.7150/ijms.50657

**Published:** 2020-09-23

**Authors:** Borja De-Yñigo-Mojado, Javier Madera-García, Ricardo Becerro-de-Bengoa-Vallejo, Marta Elena Losa-Iglesias, David Rodríguez-Sanz, Marta San-Antolín, Cesar Calvo-Lobo, Daniel López-López

**Affiliations:** 1 Facultad de Enfermería, Fisioterapia y Podología. Universidad Complutense de Madrid, Spain; 2 Department of direction, Staub Engineering, Spain; 3 Faculty of Health Sciences. Universidad Rey Juan Carlos, Spain.; 4 Department of Psychology, Universidad Europea de Madrid, Spain; 5 Research, Health and Podiatry Group. Department of Health Sciences, Faculty of Nursing and Podiatry. Universidade da Coruña, Ferrol, Spain.

**Keywords:** Community Health Workers, Filtration, Masks, Physicians

## Abstract

**Background:** Usually, physicians use filtering respirators in clinical settings to a lesser extent than other simpler surgical masks. The study aim was to determine the fit factor of surgical and other types masks commonly used in clinical settings compared with FFP3 filtering respirators.

**Materials and Methods:** A cross-sectional study was carried out recruiting a total sample of 78 physicians. Fit factor was measured to determine particles count into masks compared to particles count outside of the masks meanwhile physicians carried out a protocol composed by 8 exercises as well as the global fit factor total scores. First, fit factor was analyzed with the usual surgical masks used by physicians in clinical settings. Second, fit factor was determined with the proposed FFP3 filtering respirators.

**Results:** Most participants (97%) used surgical masks. Statistically significant differences (*P*<0.001) with an effect size from moderate to large (*d*=0.61-1.00) were shown for fit factors in the different exercises and total scores between surgical and other masks (3.2±5.0) and FFP3 filtering respirators (40.7±37.8). Generally, FFP3 filtering respirators showed a higher fit factor in the different exercises and total scores compared to the commonly used surgical and other types masks in clinical settings.

**Conclusions:** Despite most physicians used surgical masks in clinical settings, filtering FFP3 masks showed a higher fit factor in the different exercises and total scores compared with the used surgical masks and filtering respirators such as FFP1, FFP2 and other types in clinical settings.

## Introduction

Occupational exposure to physical, chemical and biological risk factors in clinical settings has received little attention. Primary care may be considered as the most common interventions performed by physicians in clinical setting. In to carry out this treatment, electric drills are used to polish the patient's nails or skin, generating large amounts of dust and organic aerosols with high infectious potential risk, which are susceptible to be inhaled by the professional who may consequently suffer from different diseases 1-3.

Centers for Disease Control and Prevention recommend the use of a filter mask for sanitary workers exposed to organic aerosols which should be previously tested by the fit factor. Nevertheless, the filtering capacity of a surgical mask seems to be poor, even if several types are simultaneously used 4-7. According to most medical specialties, public health investigations and Centers for Disease Control and Prevention have detailed unsafe practices, including medical settings patients, who could be at risk to suffer from bacterial, fungal and viral infections. Thus, all healthcare providers, such as physicians, should consider infection prevention as a priority in any clinical setting 2.

Indeed, fit factor means a quantitative estimation regarding the filtration of a particular respiration device for a specific individual. This factor estimates the concentration ratio of a substance in ambient air with respect to its concentration inside the respirator during worn. The key role of the filtering capacity of a mask is related to the ability of the mask to form a seal with the user's face, removing air leakage between the user's face and the contour of the mask 8. Currently, there are several types of fit tests that are able to measure the potential of a face seal mask to the user's face, obtaining the fit factor as a numeric value that indicates the facial fit-ability of these masks 8-11.

Generally, people are reluctant to wear masks and, specifically, physicians in particular use filtering respirators as filtering face pieces (FFP), i.e. FFP1. FFP2 and FFP3 types, to a lesser extent than other simpler surgical masks, which may allow that more particles pass into the respiratory system of physicians, likely to cause illness 2. We hypothesized that surgical masks may be the most common used mask type by physicians and these types of surgical masks and others used by physicians in clinical settings could present a lower filtration factor (fit factor) than FFP3 filtering respirators, allowing the passage of more particles which could later be inhaled by the professional. Thus, the study aim was to determine the fit factor of the surgical and other types masks commonly used by physicians in clinical settings compared with FFP3 filtering respirators.

## Material and Methods

### Study design

A cross-sectional observational study was performed at in clinical settings among physicians in the Principality of Asturias, Spain from June 2015 to December 2017, according to the Strengthening the Reporting of Observational Studies in Epidemiology recommendations *(STROBE)* 12.

### Ethical considerations

This research study was designed according to the human research principles for clinical research of the Declaration of Helsinki 13. This study was approved by the Ethics Committee of the Rey Juan Carlos University from Madrid (Spain) with a registry number code of 09/2015. Before the study beginning, all participants received full information about the study aim and procedures, as well as they signed the informed consent form.

### Sample size calculation

The sample size calculation was carried out according to the following formula:


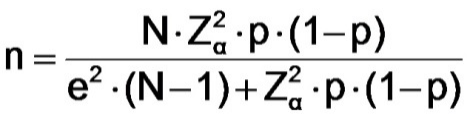


Regarding this formula, “n” was the sample size, “N” was the total population, “Z_α_” was the level of confidence, “p” was the expected ratio, and “e” was the statistic error. A 95% confidence interval (CI) according to a coefficient of Z=1.96, an expected ratio of 50% according to a p=0.05, and a desired precision of 5% according to a value of 0.05 were used for this sample size calculation. Finally, a total sample size of 78 physicians was calculated based on the total active population of physicians of N=98 at January, 2015, according to the following formula 14:

n= (98*1.962*0.5*0.5)/((0.052*97)+(1.962*0.5*0.5)) = 94,1192/1,2029 = 78,24 → n=78

### Participants

The physicians were recruited by a randomized sampling method from the total population of physicians who performed primary care in clinical settings (N=98) in the Principality of Asturias (Spain) at January, 2015 14. Inclusion criteria were physicians with an age older than 18 years from the Principality of Asturias (Spain) who carried out at least 5 primary cares per week. Exclusion criteria were physicians who suffered from pulmonary diseases, pregnant women, did not sign the informed consent form, and physicians who did not perform primary foot care according to the Occupational Safety & Health Administration (OSHA) recommendations 15.

### Descriptive data

Descriptive data, such as sex (women or men), weight (kg) measured by a digital scale (Bosch, AxxenceSlim Line model, Gerlingen, Germany), height (cm) assessed by a measuring tape (M807-20 model, Brueder Mannesmann Werkzeuge, Remscheid, Germany), face length (mm), face depth (mm), face width (mm) and mouth width (mm) measured by a compass tool (Staedtler, Mars basic 554 model, Nüremberg, Germany), were collected 8,15.

### Mask types

According to the study aim, the fit factor of the surgical and other types masks commonly used by physicians in clinical settings was determined and compared with FFP3 filtering respirators 2.

**Figure 1:**
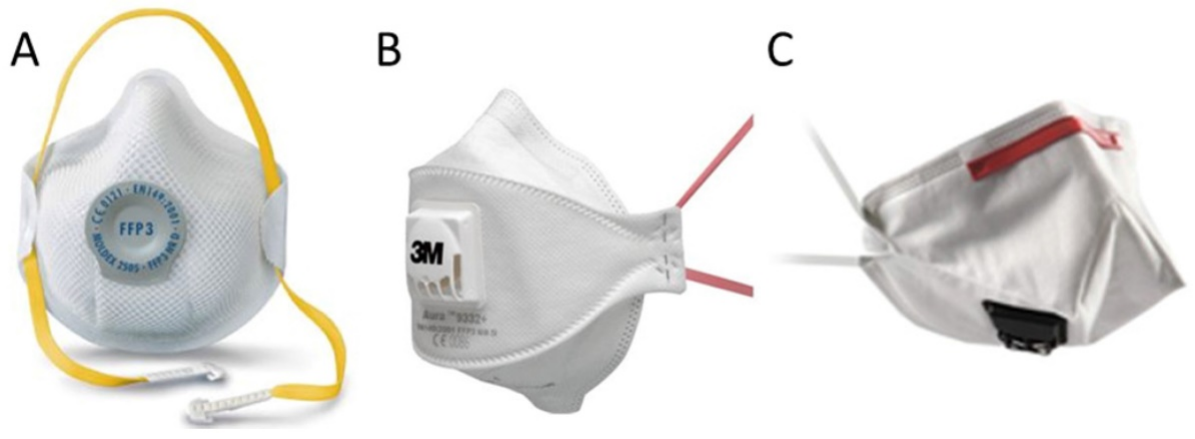
Proposed FFP3 filtering respirators models used in this study, such as Moldex 2505 (Culver City, CA, USA; Figure 1A), Aura 9332+ (3M, St Paul, MN, USA, figure 1B), and K113 (3M, St Paul, MN, USA; Figure 1C). Abbreviations: FFP, filtering face pieces.

**Figure 2:**
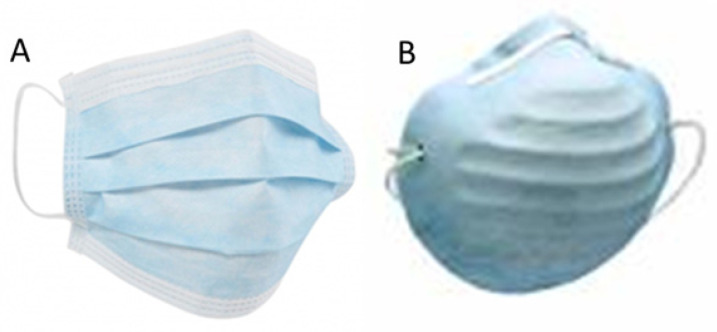
Usual surgical (Figure 2A) and other type masks (Figure 2B; i.e. Shell type).

Filtering FFP3 respirators may be considered as the most efficacious FFP masks in order to avoid virus and bacterias exposure. The proposed filtering respirator FFP3 models were Moldex 2505 (Culver City, CA, USA; Figure 1A), Aura 9332+ (3M, St Paul, MN, USA; Figure 1B), and K113 (3M, St Paul, MN, USA; Figure 1C) 8-11,16. Surgical masks (Figure 2A) and other types (i.e. Shell type; Figure 2B) were considered as the usual masks used by physicians in clinical settings 2.

### Fit factor analyses

In order to determine the count of particles which may transfer the mask of physicians in clinical settings, the fit factor of the surgical masks and filtering respirators such as FFP1, FFP2 and other types, were compared with respect to the FFP3 filtering masks 2.

According to the Occupational Safety & Health Administration (OSHA) recommendations 15, fit factor was used as the gold standard to quantify the filtration capacity of these masks. Indeed, fit factor may be defined as a quantitative estimation measurement indicating the filtration of a specific respiration device, such as a mask, for a specific individual. Specifically, this measurement determined the particles concentration ratio in ambient air compared to their concentration inside the mask during worn, detailing the ability of the mask to perform a seal within the physician´s face, avoiding air leakage between the face and the mask contour 8,17.

Following the described procedure of prior research studies 10,18-21 and OSHA protocol 15, fit factor was used as a quantitative method to determine particles count into masks compared to particles count outside of the masks meanwhile physicians carried out a protocol composed by 8 exercises (Table 1). First, fit factor was analyzed with the usual surgical masks and filtering respirators such as FFP1, FFP2 and other types. Second, fit factor was determined with the proposed FFP3 filtering masks (Figure 1).

**Figure 3:**
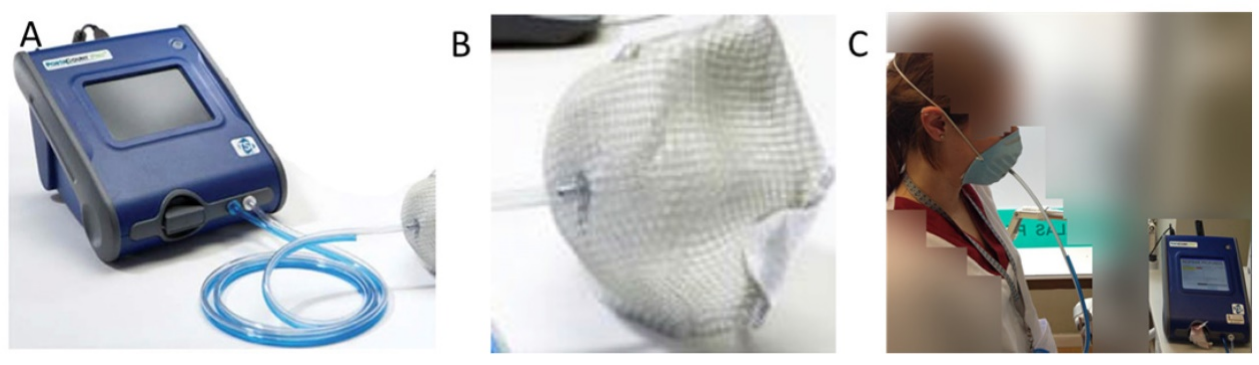
Tool for fit factor analyses (PortaCount® Pro+ Modelo 8038; Figure 2A), mask (Figure 2B) and leak-proof kit of catheters and adapters (TSI, Tsi Inc, St Paul, MN, USA: Figure 2C).

For these fit factor analyses, the adjusted quantitative analyses were performed by a reliable tool 22, called PortaCount® Pro+ Modelo 8038 (Figure 3A), which presented CE certificate and previously calibrated. According to the technical characteristics of this tool, this model measured particles with a size rank from 0.02 to >1μm. From this tool, 2 catheters were provided and the longest catheter was connected to the mask (Figure 3B) by a leak-proof kit of catheters and adapters (TSI, Tsi Inc, St Paul, MN, USA; Figure 3C). Thus, the probe of the catheter was located between the nose and mouth at 5 mm from the interior surface of the mask and 10-15 mm from the physicians' mouth, containing an air sample inside of the mask. In addition, all measurements were carried out in a clean room of a surface of 15 m^2^, approximately 19,23.

According to prior studies 10,18-21 and OSHA protocol 15, total fit factor was calculated by the global fit factor adjustment as a pondered mean of the 8 exercises in relation to the particles count that a physician could inhale in the primary care service, by the following formula considering “N” was the number of performed exercises and “FFn” was the fit factor obtained for a specific exercise number:

Fit factor total (FFT) = N / [(1/FF1)+(1/FF2)+(1/FF3)+ … +(1/FFn-1)+(1/FFn)]

### Statistical analyses

Statistical analyses were performed using 23.0 version of the SPSS software (IBM SPSS Statistics; Windows; IBM Corp; Armonk-NY, USA). For all analyses, a *P*-value* <* 0.05 for a 95% confidence interval (CI) was considered as statistically significant.

For quantitative data, normality analyses were performed by the Kolmogorov-Smirnov test. After, these data were described as mean ± standard deviation (SD), median as well as lower and upper limits for a 95% CI. Next, comparisons for fit factor of each exercise and global fit factor between usual surgical masks and the proposed filtering masks were carried out by the Student's t-test for related samples. Box-plots were used in order to illustrate fit factor 95% CI comparisons for fit factor of each exercise (from 1 to 8) and global fit factor (T) between surgical and filtering masks. In addition, effect sizes for these comparisons were analyzed by the Cohen's *d* calculated by the following formula *d* = (*M*_2_ - *M*_1_) ⁄ *SD*_pooled_ using the mean difference between both groups divided by the pooled SD 24. These values were categorized as small (*d*<0.20), small (*d*=0.20-0.49), medium (*d*=0.50-0.79), and large (*d*>0.8) effect sizes 25. For categorical data, frequencies (n) and percentages (%) were used to describe these data.

## Results

### Descriptive data

From the total sample of 78 participants, 47.4% (n = 37) were men and 52.6% (n = 41) were women. All participants showed an age range from 22 to 62 years with an age mean ± SD (95% CI) of 34.3 ± 7.1 (32.7 - 35.9) years. Table 2 showed descriptive data including mean, SD, lower and upper limits of the 95% CI values of the study sample.

### Mask types

According to Table 3, most participants (97%) used masks different from FFP1, FFP2 or FFP3. Surgical masks and filtering respirators such as FFP1, FFP2 and other types, were the most used (97.4%) in clinical settings, while FFP3 filtering masks were only used by the 2.6% of physicians.

### Fit factor comparisons

**Figure 4:**
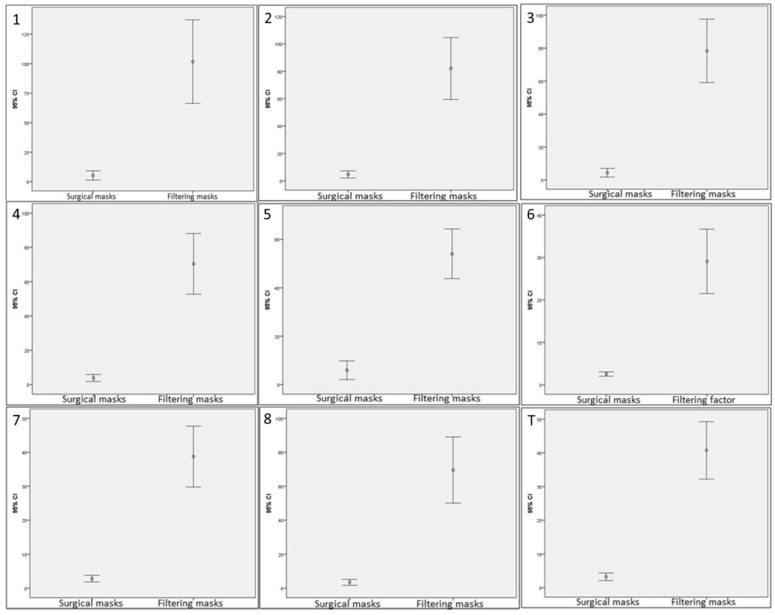
Box-plots to illustrate fit factor 95% confidence intervals comparisons for exercises (from 1 to 8) and total scores (T) between surgical and other types masks with respect to FFP3 filtering respirators used by physicians in primary care. Abbreviations: FFP, filtering face pieces.

Regarding Table 4 and Figure 4, statistically significant differences (*P* < 0.001) with an effect size from moderate to large (*d* = 0.61 - 1.00) were shown for fit factors in the different exercises and total scores between surgical and other types with respect to FFP3 filtering masks. Generally, FFP3 filtering respirators showed a higher fit factor in the different exercises and total scores compared with the used surgical masks and filtering respirators such as FFP1, FFP2 and other types in clinical settings.

## Discussion

Infection prevention and control remain as a challenge to provide consistently safe care in clinical settings 2. To the best of our knowledge, this study may be considered as the first descriptive research detailing the current use of surgical and other masks among physicians in clinical settings. Furthermore, fit factor of these commonly used masks was compared with the recommended FFP3 filtering respirators in order to avoid exposure to virus and bacteria, among others 8-11,16.

According to our study findings, the 97% of physicians used masks different from FFP1, FFP2 or FFP3. In addition, surgical and other masks were used for the 97.4% of physicians and filtering masks were only used by the 2.6% of physicians. This high percentage of masks use with respect to the low frequency for the use of filtering respirators among physicians may be partially explained due to this study was carried out before beginning the COVID-19 pandemic 26. Indeed, fit factors for each proposed exercise and global fit factor were clearly superior for filtering FFP3 masks with 40.7±37.8 points compared to the usual surgical and other types masks used in clinical settings with 3.2±5.0, being FFP3 filtering masks global fit factor about more than 12 times greater than surgical and other types masks global fit factor. Our findings were in line with prior studies 10,18-21 and OSHA recommendations 15, thus, we encourage physicians to use filtering FFP3 mask during primary care service.

Nevertheless, prior research studies have reported lower proportions than those observed in our study related to the installation of mechanical ventilation systems. A study carried out in Ireland on 101 physicians found that only a proportion of 15.8% used mechanical ventilation in the work room, while 47.5% used micro-motors with exhaustive local ventilation systems and 11% micro-motors with jet water for particle suppression 27.

According to a randomized controlled pilot study 28, all subjects using the FFP2 mask type should achieve a filtration factor of ≥ 100, as they had previously passed a fit test with the same mask model. The lower than expected filtration rate may reflect the fact that subjects did not undergo a regular fit program, so it is recommended that workers wearing FFP2 masks perform repeated fit tests on a regular basis, despite that may suppose a considerable logistical and financial burden. Nevertheless, the data suggests that a significant proportion of subjects may not be adequately protected without this type of regular fit testing 29.

Prior studies revealed the exposure of physicians to pathogenic microorganisms related to nail dust. In order to determine air routes and microbial species, surface air sampling studies and a variety of crops have been conducted. Isolated fungal microorganisms in the air were identified. Dermatophyte fungi have been approximately collected in 80-90% of all nail infections, i.e. onychomycosis 30. Trichophyton Rubrum, which was considered as an organism that may cause bronchial asthma, was associated with other symptoms, such as rhinitis or allergic hypersensitivity 31. A prior research has found that physicians may show antibodies against this organism, suggesting routine exposure to it 32. Other authors showed the benefits of an air filtration system, reducing up to 60% of global clinical air pollutants 33. In this sense, scientific evidence supports the theory of primary care occupational risk of respiratory health from environmental dust. A risk reduction strategy may be the use of masks with an adequate protection factor against these pathogens, which could effectively reduce the levels of exposure to nail dust.

### Limitations and future research

Some limitations should be considered in the present study. First, this study followed an observational research design. Future single blinded randomized clinical trials should compare the fit factor effectiveness of filtering masks versus surgical masks during primary care by physicians, being especially relevant during the current COVID-19 pandemic 26. Second, this study was carried out according to a sample size calculation based on the total population of physicians who performed primary care in the Principality of Asturias (Spain). Nevertheless, we should consider a sample size calculation based on the total population of physicians in Spain, as well sample size calculations for interventional studies according to pilot randomized clinical trials.

## Conclusions

Despite most physicians used surgical mask in clinical settings, filtering FFP3 masks showed a higher fit factor in the different exercises and total scores compared with the used surgical masks and filtering respirators such as FFP1, FFP2 and other types in these clinical settings. Future randomized clinical trials should be carried out in order to promote filtering masks use among physicians who perform primary care service.

## Figures and Tables

**Table 1:** Exercises protocol to determine the fit factor

Exercise number	Exercise name	Exercise description (1 minute per exercise, except exercise number 6 for 15 seconds)
Exercise 1	Normal breathing	Physicians was quite with usual breathing
Exercise 2	Deep breathing	Physicians carried out deep and large respirations like performing a great effort
Exercise 3	Neck side bending	Normal breathing meanwhile the neck was side bending and neck muscles were stretched
Exercise 4	Speaking out loud	Physicians was speaking out loud counting from zero
Exercise 5	Head flexion and extension	Normal breathing during head flexion and extension
Exercise 6	Grimaces	Physicians smiled or frowned up to 15 seconds
Exercise 7	Trunk flexion	Physicians performed a trunk flexion touching their toes
Exercise 8	Normal breathing	Similar to exercise 1, physicians was quite with usual breathing

**Table 2:** Descriptive data of the study sample (n=78)

	Mean (SD)	95% CI limits	
		Lower	Upper
Weight (kg)	69.6 (12.5)	66.8	72.3
Height (cm)	169.3 (8.9)	167.4	171.3
Face length (mm)	112.5 (9.0)	110.5	114.5
Face depth (mm)	122.9 (8.2)	121.1	124.7
Face width (mm)	134.1 (8.2)	132.2	135.9
Mouth width (mm)	48.4 (4.2)	47.5	49.3

Abbreviations: CI, confidence interval; SD, standard deviation

**Table 3:** Mask type used by physicians in clinical settings (n=78).

Masks and respirators		Type	Physicians (n)	Percentage (%)
Surgical and other masks		Surgical and others	71	91%
		FFP1	2	2.6%
		FFP2	3	3.8%
Filtering respirators		FFP3	2	2.6%

Abbreviations: FFP, filtering face pieces.

**Table 4:** Fit factor comparisons for exercises and total scores between surgical and other masks versus FFP3 filtering respirators.

	Surgical and other masks (n = 78)					FFP3 filtering respirators (n = 78)					Surgical vs FFP3 filtering respirators*		
	Mean	SD	Median	95% CI limits		Mean	SD	Median	95% CI limits		*t* (77)	*P*	*d*
				Lower	Upper				Lower	Upper			
Exercise 1	5.3	17.5	2.1	1.4	9.2	101.7	157.0	42.0	66.9	136.6	-5.381	<0.001	0.61
Exercise 2	4.7	11.7	2.2	2.1	7.3	82.1	100.1	46.0	59.9	104.3	-6.783	<0.001	0.77
Exercise 3	4.4	11.6	2.1	1.8	7.0	78.4	85.5	51.5	59.4	97.3	-7.492	<0.001	0.85
Exercise 4	3.9	9.1	2.1	1.9	5.9	70.4	78.5	42.0	53.0	87.8	-7.402	<0.001	0.84
Exercise 5	5.9	17.1	2.7	2.1	9.7	54.0	45.5	39.0	43.9	64.1	-8.633	<0.001	0.98
Exercise 6	2.6	2.3	2.0	2.1	3.1	29.1	33.6	16.5	21.6	36.6	-7.070	<0.001	0.81
Exercise 7	2.8	4.3	1.9	1.9	3.8	38.7	39.8	24.5	29.9	47.6	-7.960	<0.001	0.91
Exercise 8	3.5	7.7	2.0	1.8	5.2	69.6	86.5	47.0	50.4	88.8	-6.739	<0.001	0.77
Total score	3.2	5.0	2.1	2.1	4.3	40.7	37.8	28.5	32.3	49.1	-8.733	<0.001	1.00

Abbreviations: CI, confidence interval; FFP, filtering face pieces; SD, standard deviation; *t*, Student *t* statistic for a 95% CI (degrees of freedom); *d*, Cohen *d* statistic for effect size. *Student t test for related samples were applied for a *P* < 0.05 as statistically significant for a 95% CI.
